# Application Value of Magnetic Resonance Perfusion Imaging in the Early Diagnosis of Rat Hepatic Fibrosis

**DOI:** 10.1155/2019/5095934

**Published:** 2019-12-28

**Authors:** Guohua Fan, Yang Ya, Xiaoqiong Ni, Jinpeng Hou, Rui Yu

**Affiliations:** Department of Radiology, The Second Affiliated Hospital of Soochow University, Suzhou 215004, China

## Abstract

**Objective:**

To assess the application value of perfusion-weighted imaging (PWI) in early diagnosis, quantitation, and hepatic fibrosis staging by analyzing the related parameters in hepatic fibrosis.

**Methods:**

A total of 60 rats were randomly divided into the hepatic fibrosis and control groups, and carbon tetrachloride (CCL4) was used to establish the liver fibrosis model. All rats underwent PWI examination, and the trend of the time-signal intensity curve (TIC, automatically generated by the software) was observed. Also, the perfusion parameters, maximum signal reduction ratio (SRRmax), time to peak (TTP), and mean transit time (MTT), were analyzed and compared with pathological staging.

**Results:**

The TIC curve was characterized by slow wash-in and wash-out with a low and wide peak. The PWI perfusion parameters were statistically significant in specific groups (*P* < 0.05): SRRmax values (control group and F3, F4), TTP, and MTT values (control group and F2–F4, F1 and F3, F1 and F4, and F2 and F4 in addition to TTP values for F1 and F2). Pearson's correlation analysis showed a negative correlation of SRRmax with hepatic fibrosis stage (*r* = −0.439, *P* < 0.05), while TTP and MTT values were positively correlated with hepatic fibrosis stage (TTP, *r* = 0.798; MTT, *r* = 0.647; all *P* < 0.001).

**Conclusions:**

PWI perfusion parameters reflect the degree of hepatic fibrosis, especially TTP and MTT, and PWI is recommended for the early diagnosis of liver fibrosis for timely intervention and treatment of the disease and delaying its progression.

## 1. Introduction

Hepatic fibrosis is a life-threatening disease with high morbidity and mortality owing to its diverse causes. It is characterized by excessive deposition of collagen extracellular matrix (ECM) components, which support the liver [1]. Hepatic stellate cells (HSCs) are the main source of collagen and ECM [2]. After liver injury, resting HSCs are transformed into activated myofibroblasts, followed by ECM synthesis [3]. When the synthesis of ECM is greater than consumption, hepatic fibrosis develops gradually, leading to liver dysfunction, portal hypertension, and hepatocellular carcinoma (HCC) [1, 4].

Presently, the early detection of liver fibrosis is unclear, and hence, finding a method for the diagnosis of the disease is an urgent requisite. Perfusion-weighted imaging (PWI) is a noninvasive method for assessing microvascular tissue distribution, viability, and function via tissue microcirculation hemodynamics, which is superior to traditional magnetic resonance imaging (MRI) [5, 6]. Previously, the clinical application of PWI in thoracic and abdominal organs was limited due to physiological movements [5, 7]. In recent years, with the development and application of echo-planar imaging (EPI), the influence of physiological activity on signal acquisition is greatly reduced, which lays a foundation for the widespread application of PWI in liver lesions.

Previous studies [8–10] on PWI perfusion parameters have shown that patients with advanced hepatic fibrosis have significantly higher MTT and TTP, increased hepatic arterial perfusion, and decreased portal perfusion as compared with patients with nonprogressive hepatic fibrosis, suggesting that PWI exerts a positive effect on the early diagnosis of liver fibrosis. Furthermore, perfusion imaging has shown increasing potential in this field and is expected to be an effective method for analyzing the liver function and determining the nature of liver lesions [11–13].

The present study aimed to explore the application value of noninvasive PWI in early diagnosis, quantization, and staging of hepatic fibrosis by analyzing the PWI perfusion parameters (SRRmax, TTP, and MTT).

## 2. Materials and Methods

All experimental animal protocols were in accordance with the Guide for the Care and Use of Laboratory Animals and approved by the Animal Experimental Ethics Committee of the Second Affiliated Hospital of Soochow University (Suzhou, China). Efforts were made to minimize the suffering of the animals and reduce the number of animals used.

### 2.1. Liver Fibrosis Model

A total of 60 male, 6-week-old, Sprague Dawley rats, weighing 180–200 g, were purchased from the Experimental Animal Center of Soochow University and randomly divided into the hepatic fibrosis group (*n* = 48) and the control group (*n* = 12). In the hepatic fibrosis group, 40% CCL4 oil solution was injected subcutaneously (initial dose was 5 mL/kg, followed by 3 mL/kg, twice a week, intraperitoneally). 10% ethanol solution was the only drinking liquid, which established a composite liver fibrosis model combining chemical and alcohol factors to shorten the modeling time and improve the success rate. The control group was intraperitoneally injected with normal saline, and the dosage and usage were identical to that of the hepatic fibrosis group, and purified water was the drinking liquid. The prolonged modeling time led to the loss of appetite, mental collapse, decreased activity, weight loss, malnutrition, and hairlessness in the hepatic fibrosis group, while the rats in the control group showed bright hair and an increase in the body weight.

### 2.2. MR Perfusion Imaging

From the 2nd week after modeling, in order to maintain a relatively balanced number of rats between the groups, 4 model and 1 control rats were randomly selected every week for scanning; the remaining model rats were continuously injected with CCL4, and the abovementioned steps were repeated until the end of the experiment at week 10. MRI scan was performed using a multichannel whole-body MR scanner (Philips Achieva System, the Netherlands), with a field strength of 1.5 T and a bore size of 60 cm. About 10–30 min before the scan, the rats were anesthetized by 2% pentobarbital sodium (40 mg/kg, intraperitoneally). Then, the animals were fixed on the gantry (in the prone position) with the liver placed in the center of the 4.7 cm microcoil of the microscope (Philips). Abdominal compression bandages were applied to restrain the respiratory movement range, and the height of the gantry was adjusted before scanning to ensure that the rat liver was at the center of the magnet. First, the three planes of the positioning image were scanned, followed by the conventional turbo spin-echo (TSE) sequence of the axial T2WI-SPAIR and T1WI scan, and finally the PWI scan was started. After scanning five dynamics, the magnetic contrast agent (Gd-BOPTA, commodity name MultiHance, containing 529 mg of gadobenate dimeglumine/mL (gadolinium acid 334 mg + meglumine 195 mg)) was rapidly injected into the rat tail vein, at a dose of 0.2 mmol/kg (the average injection volume was about 0.5 mL per rat) at a flow rate of 2 mL/s, and 40 consecutive dynamic scans were performed at a scanning time of 97 s, and 8 to 12 layers covered the whole liver. PWI uses a single-shot spin-echo echo-planar imaging (SE-EPI) and fat suppression techniques to exclude the artifacts due to the chemical shifts.

### 2.3. Histological Examination

Within 4 h after PWI scanning, the rats were decapitated according to the ethical requirements of the animal experiment. Based on the liver function analysis of the MR region of interests (ROIs), the right liver lobe was fixed with 4% formaldehyde, embedded in paraffin, and stained with hematoxylin-eosin (H&E), Masson trichrome, and reticular fiber. All slices of the fixed liver (a minimum of two (1 mm × 1 mm × 1 mm) from each rat) were examined by using a light microscope (Olympus CH30, Tokyo, Japan). Based on the histological manifestations, the METAVIR-scoring system was used to evaluate the liver fibrosis stage on a scale of 0–4: F0: no fibrosis; F1: collect abbacy fibrosis, no fibrous septa; F2: a small amount of fibrous septa is formed; F3: excessive fibers formed between deformed lobular structures; F4: liver cirrhosis [14]. Ultrastructural changes of the liver tissue were observed by transmission electron microscopy (Hitachi H-600, Tokyo, Japan), including hepatocyte morphology, nucleus, organelle changes, sinusoidal morphology, endothelial cell changes, Disse space, and fibrous tissue proliferation in the hepatic lobule. All histological examinations were undertaken by an experienced pathologist blinded to the study protocol.

### 2.4. Image Analysis

Perfusion software (Philips, the Netherlands) was used to process the original image. The time-signal intensity curve (TIC) was automatically generated by the software to analyze the signal intensity changes of ROIs at different time points. Notably, ROI included the entire liver but avoided its large blood vessels and was manually plotted in the original image. The perfusion parameters were calculated as follows: (1) maximum signal reduction ratio (SRRmax): SRRmax = (SI_pre_ − SI_p_)/SI_pre_ × 100%, where SI_pre_ is the average value of the signal intensity of ROIs after perfusion scan image is stabilized and before the signal declines, and SI_p_ is the value at which ROI signal intensity drops to its peak. (2) Time to peak (TTP) : the time from the point when the ROI signal intensity begins to decrease (*t*_0_) to the time when the drop reaches the peak (*t*_p_). (3) Mean transit time (MTT): the time for the contrast agent to pass through the ROI capillary network, MTT=∫*t*Δ*R*2^*∗*^d*t*/∫Δ*R*2^*∗*^d*t*, Δ*R*2^*∗*^=−{ln(*S*_*t*_/*S*_0_)}/*TE*, where *S*_0_ is the image signal intensity on the precontrast images, *S*_t_ is the image signal intensity measured at time *t*, time-relaxation rate (Δ*R*2^*∗*^) curve is proportional to the relative blood flow [15]. Finally, Pearson's correlation between perfusion parameters and pathological stages of hepatic fibrosis was analyzed.

### 2.5. Statistical Analysis

All data showed normal distribution and were expressed as mean ± standard deviation. Single-factor analysis of variance (one-way ANOVA) was used to compare the differences between groups, followed by mean multiple comparison analysis of each group. For all pairwise group comparisons, *t*-test and Duncan' test were used for equal variance, and a two-tailed Student's *t*-test was used for unequal variances. All pairwise correlations between variables were examined by calculating Pearson's correlation coefficient. *P* < 0.05 indicated a statistically significant difference. The analyses were performed using SPSS, version 16.0 (Chicago, IL, USA).

## 3. Results

### 3.1. Establishment of Rat Liver Fibrosis Model

A total of 17 rats that did not meet the experimental requirements (death or imaging failure) were excluded. The causes of death in rats included the toxic effects of CCL4 during modeling: excessive anesthesia or anesthetic is accidentally injected into the blood vessel; the abdominal compression bandage is extremely tight, affecting the respiratory movement of rats; and rats with advanced liver fibrosis had abnormal liver function and decreased tolerance to anesthetic agents. The main reasons of perfusion imaging failure included failure of tail vein puncture, external leakage during contrast injection, unsatisfactory anesthesia affect imaging, and poor image quality impact analysis.

**Figure 1:**
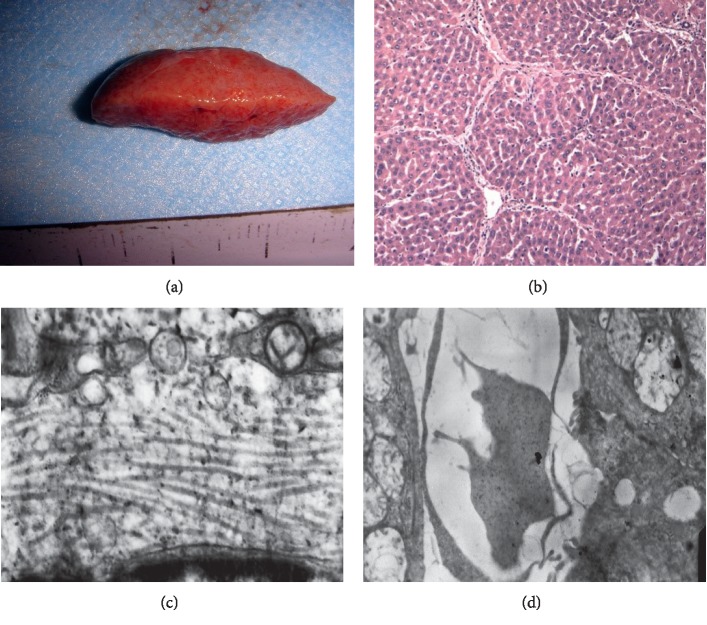
Gross specimen of the liver assessed by HE staining and transmission electron microscopy: (a) liver fibrosis specimens show that the liver surface and edge are not smooth; (b) hematoxylin-eosin (×100) and fibrous septa are formed; (c) transmission electron microscopy (×12000) shows thickening of the fibrous tissue septa, and deposition of the collagen fibers; (d) transmission electron microscopy (×12000) shows a decreased number of fenestrations of liver sinusoidal endothelial cells, and incomplete basement membrane formation was observed subcutaneously, which occurred during the hepatic sinus capillarization.

**Figure 2:**
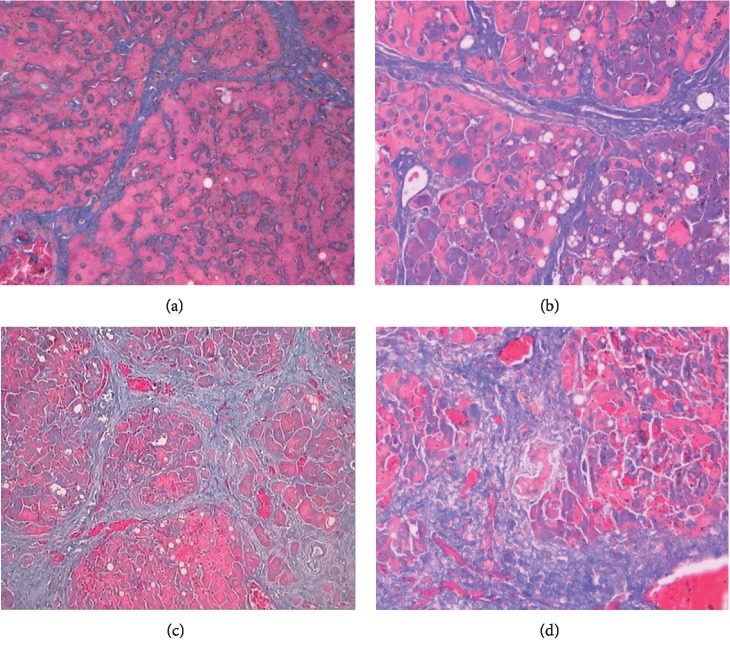
Different degrees of liver fibrosis by Masson trichrome staining (×200): (a) F1, a few fibrous strips formed; (b) F2, a large number of dense intrahepatic fiber cords than F1; (c) F3, the number of fiber cords is further increased; (d) F4, a large number of fiber cords accumulate, and the hepatic lobule structure is disordered.

**Figure 3:**
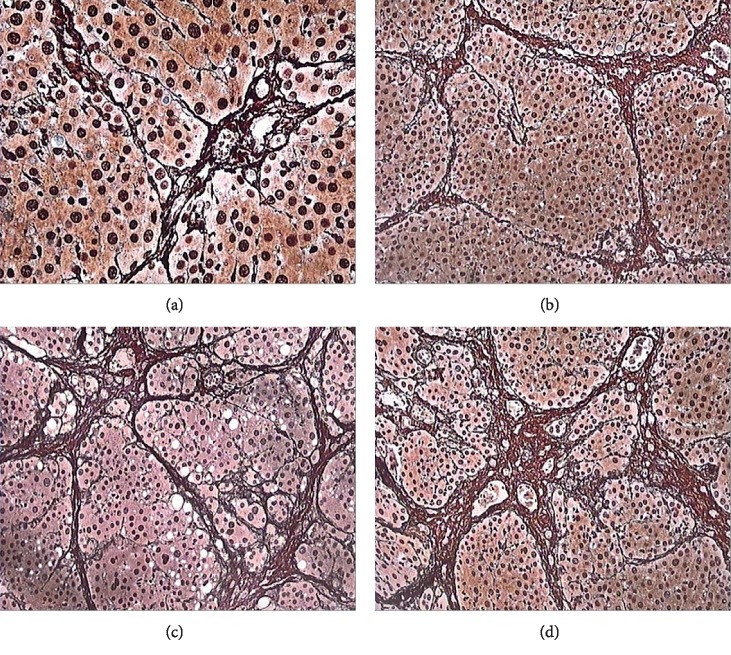
Different degrees of liver fibrosis reticular fiber staining: (a) F1, cumulative abbacy fibrosis (×400); (b) F2, fibrous septa accompanied by liver steatosis (×200); (c) F3, fibrous septa formed and disordered lobular structures (×200); (d) F4, a large number of collagen fibers/reticular fibers and formation of pseudolobe (×200).

Liver fibrosis was not observed in the control group rats (F0, *n* = 8), while all tetrachloride-injected rats developed liver fibrosis (model, *n* = 35). F1 fibrosis was observed in seven rats, F2 in nine, F3 in eleven, and F4 in eight rats. Early fibrosis was characterized by fibrosis in the portal area and a small number of fiber cords; as the degree of fibrosis deepens, the number of fiber strands increases, the distribution becomes disordered, and pseudolobules are formed (Figures 1–3).

### 3.2. Liver Fibrosis PWI Performance

**Figure 4:**
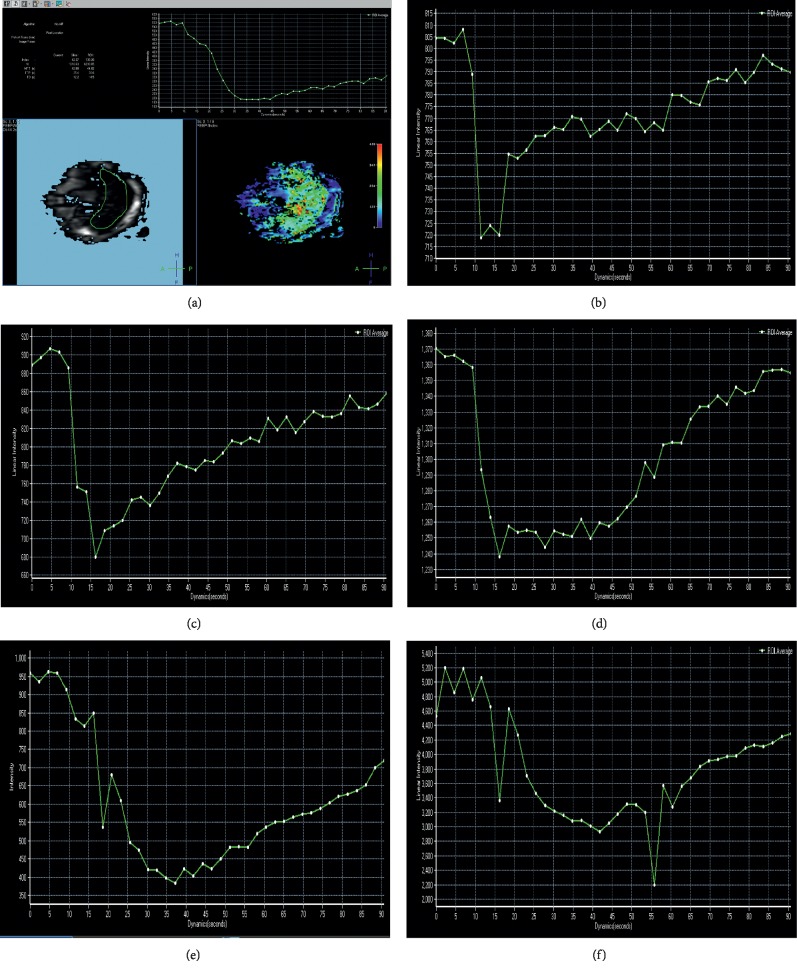
TIC curves of normal and various stages of liver fibrosis: (a) PWI parameters analysis; (b) TIC curve in the control group; and (c–f) TIC curve in F1–F4.

The liver perfusion baseline of 43 rats was smooth, and the morphology of liver parenchyma perfusion curve was stable, showing different trends due to the degree of fibrosis (Figure 4). The dynamic variation characteristics of perfusion imaging signals were as follows: after the contrast agent was injected, the intensity of the liver signal decreased and then recovered slowly; also, the TIC curve of the liver parenchyma showed a corresponding change in the trend. Interestingly, the TIC curve was characterized by slow wash-in and slow wash-out with a low and wide peak. In the control group, the curve showed a rapid decline, followed by slow recovery after reaching the peak, with a large recovery range and a short recovery time (Figure 4(b)). Conversely, the curve of the hepatic fibrosis group descended slowly and the amplitude decreased, which prolonged the time to reach the peak. After reaching the peak, the recovery range was small, the recovery time was long, the wave peak was wide, and the enhancement of the liver parenchymal increased slowly (Figures 4(c)–4(f)). Notably, this change was pronounced with increasing stage of liver fibrosis (Figures 4(b)–4(f)).

### 3.3. Analysis of Liver Perfusion Parameters

**Figure 5:**
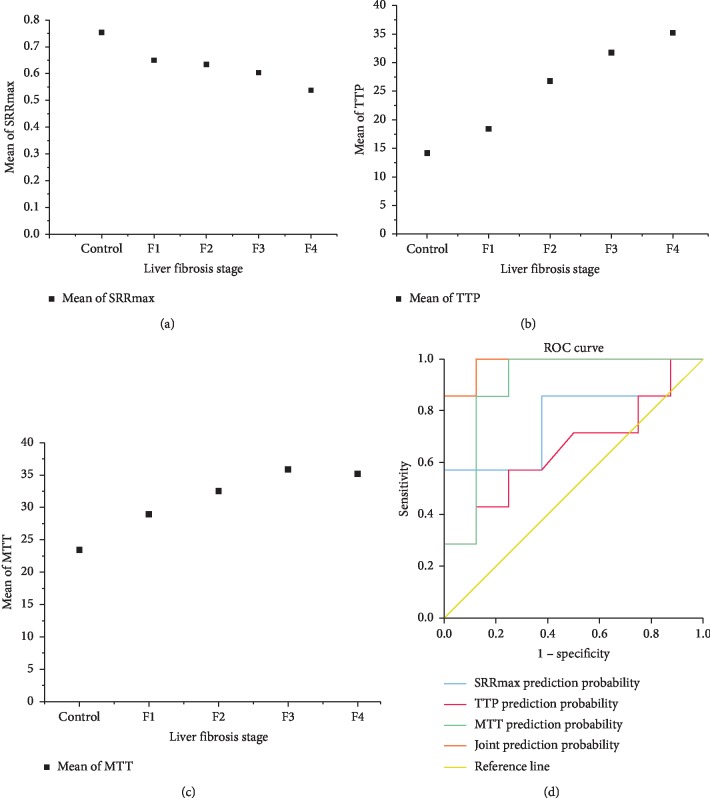
Statistical charts associated with PWI perfusion parameters: (a) the scatter plot of liver fibrosis stage and SRRmax; (b) the scatter plot of liver fibrosis stage and TTP; (c) the scatter plot of liver fibrosis stage and MTT; (d) the ROC curve of PWI perfusion parameters for F0 and F1.

As the degree of liver fibrosis increased, the SRRmax value decreased, while TTP and MTT values increased gradually (Table 1); these parameters were statistically significant. Pearson's correlation analysis showed that SRRmax was negatively correlated with liver fibrosis stage, while TTP and MTT values were positively correlated with liver fibrosis stage (Table 2). The scatter plots of liver fibrosis stage and mean perfusion parameters (Figures 5(a)–5(c)) and the receiver operating characteristic (ROC) curve of PWI perfusion parameters for F0 and F1 (Figure 5(d)) allowed us to conclude that compared with a single parameter, and the combination of the three parameters significantly improves the detection efficiency of F1.

#### 3.3.1. SRRmax Statistical Analysis

Statistically significant differences were detected between the control group and F3 and F4 (*P* < 0.05), while no significant difference was observed between the other groups (*P* > 0.05).

#### 3.3.2. TTP Statistical Analysis

Statistically significant differences were observed between the control group and F2–F4, F1 and F2, F1 and F3, F1 and F4, and F2 and F4 (*P* < 0.05). Also, no significant difference was detected between the control group and F1, F2 and F3, and F3 and F4 (*P* > 0.05).

#### 3.3.3. MTT Statistical Analysis

Statistically significant differences were observed between the control group and F2–F4, F1 and F3, and F1 and F4 (*P* < 0.05), while no significant difference was detected between the control group and F1, F1 and F2, F2 and F3, F2 and F4, and F3 and F4 (*P* > 0.05).

## 4. Discussion

The current study demonstrated that SRRmax, MTT, and TTP were altered in rats with varying degrees of hepatic fibrosis, and as the degree of hepatic fibrosis increased, the SRRmax value decreased, while that of TTP and MTT increased. PWI has different discriminating ability in the different stages of hepatic fibrosis. These three perfusion parameters, especially TTP and MTT, can detect early fibrosis, which is helpful for clinical early diagnosis and intervention, thus delaying the progression of fibrosis.

Hepatic sinus is equivalent to the capillaries of the liver. A variety of pathogenic factors lead to changes in the morphology and function of the sinusoidal endothelial cells; for example, the fenestrae gradually shrink or even disappear, the subcutaneous basement membrane is formed, and continuous capillaries, known as sinusoid capillarization is observed [16, 17]. The sinusoidal endothelium cells play a pivotal role in hepatic fibrosis and are the main factors affecting liver microcirculation hemodynamics. Hepatic sinusoid capillarization prevents oxygen diffusion into the Disse space, leading to ischemia, hypoxia, and activation of stellate cells [18, 19]. Activated HSCs synthesize ECM and produce abundant fibrous connective tissue, leading to compression and stenosis of liver sinus and portal vein branches, increased blood flow resistance of hepatic sinus and portal pressure, and decreased portal venous perfusion, which is the so-called hemodynamic change [20, 21].

In the hepatic fibrosis group (*n* = 35), light microscopy showed fibrous connective tissue hyperplasia around the hepatic sinus, central vein, and portal vein; transmission electron microscopy showed a decrease in the number of sinusoidal endothelial cells, subcutaneous basement membrane formation, hepatic sinus capillary vascularization, deformation, and stenosis. With the increase in hepatic fibrosis stage, PWI showed a decrease in liver parenchymal enhancement rate, and the TIC curve morphology and perfusion parameters changed; this phenomenon was based on the pathological changes.

Perfusion is the microscopic movement of the capillary bed, which refers to the process of blood perfusion from the artery to the capillary network, and then into the vein, which reflects the duration of the local tissue blood flow [22]. Currently, EPI is the rapid developing MRI technology with high temporal resolution [23, 24]. It can evaluate the organization and hemodynamic perfusion before the contrast agent travels from the intravascular to extracellular space. Moreover, it is viewed as a noninvasive test method to observe the hemodynamic changes of tissue microcirculation and assess the vitality and function of the organization. MR perfusion imaging is more sensitive than CT perfusion imaging without any ionizing radiation-induced damage. In addition, MRI can perfuse the whole liver, providing a more comprehensive and accurate information of the blood supply than partial perfusion of multidetector computed tomography (MDCT) [25].

Presently, liver MR perfusion imaging and perfusion models are yet in the exploratory stage with constant improvement [21, 26]. Based on the different principles of magnetic resonance perfusion imaging, three methods have been devised [27]: contrast agent first-pass, arterial spin-labeling, and blood oxygen level-dependent contrast enhancement technology. Hitherto, contrast agent first-pass is the most widely used imaging method [28]. The basic principle is as follows: when the diffuse contrast medium is rapidly injected into the bloodstream in a short period and passes through the capillary network in an area of tissue, it increases the inhomogeneity of the local magnetic field and creates a difference in the magnetic susceptibility between the local and surrounding tissues. Consequently, the T1 and T2 (relaxation rate effect) or the T2^*∗*^ value (magnetic susceptibility effect) of the local blood flow is decreased, especially the T2 relaxation time, thereby altering the intensity of the local tissue signal. The gamma function fitting can be used to eliminate the influence of contrast agent circulation and leakage effects on the quantitative perfusion analysis [15]; thus, the changes in the signal of the contrast agent in the first pass reflect the status of tissue perfusion and indirectly reflect the microvascular distribution of the organization. The quantitative and semiquantitative evaluation of the local tissue perfusion parameters is based on the changes in microcirculation and hemodynamics of the liver lesions [8, 26, 29].

GD-BOPTA provides information on the vascular changes and hepatocyte function, increasing the detection sensitivity of early HCC to 91–93% [30]. The relaxation rate of GD-BOPTA is significantly higher than that of the other types of gadolinium paramagnetic contrast agents; it is twice that of GD-DTPA, and even half dose can achieve the same strengthening effect [31]. The present study used the GD-BOPTA efficiency and T2^*∗*^ negative enhancements on T2WI liver perfusion. The advantages were as follows: (1) high relaxation rate, sensitive to changes in blood flow; (2) high temporal resolution; and (3) perform a high level, continuous multiphase scanning, and complete the entire liver perfusion imaging. As liver imaging is susceptible to artifacts (magnetic-sensitive and chemical shift), it is necessary to ensure the stability of the gradient field, optimize the imaging parameters, and use fat suppression techniques to obtain a satisfactory liver perfusion image.

Based on the results of this study, PWI can detect early liver fibrosis, provide early diagnosis for clinical practice, and apply effective treatment before the disease progresses to liver cirrhosis or cancer in order to prolong the survival of patients. The MR perfusion imaging is the preferred method while investigating cirrhosis. Some studies [9, 32] demonstrated that patients with cirrhosis had lower blood volume and blood flow than normal individuals; however, the mean transit time was prolonged. Frank et al. [33, 34] studied the portal vein blood perfusion of liver cirrhosis in animal models and found that the TTP value of liver cirrhosis perfusion curve was positively correlated with the liver cirrhosis pathological grading, and PWI was expected to find liver fibrosis at an early stage. Another study [35] suggested that hepatic interlobular perfusion was heterogeneous, in which the left lateral liver lobe (LLL), the left medial liver lobe (LML), the right liver lobe (RL), the caudate lobe (CL), and TTP were positively correlated with the hepatic fibrosis stage, while positive enhancement integral (PEI) and maximum slope of increase (MSI) were negatively correlated. The abovementioned studies indicated that MR perfusion imaging could be used to detect and stage the early liver fibrosis based on the degree of change in the perfusion parameters.

The study of MR blood perfusion in liver fibrosis is still in the exploratory stage. Hagiwara et al. demonstrated that patients with advanced liver fibrosis had increased hepatic arterial blood flow, arteriolar fraction, the volume of distribution, and MTT, but the portal vein scores declined [8, 36, 37]. Chen et al. [38] reported that the slope of time-signal intensity curves and the area under the curve declines gradually with the increase in liver fibrosis stage; these perfusion parameters could evaluate the degree of liver fibrosis adequately. In addition to changes in MTT and TTP, some studies reported abnormalities in intrahepatic perfusion in patients with progressive hepatic fibrosis; consequently, portal vein fraction decreased, the hepatic artery enhancement fraction increased, the hepatic artery perfusion value (HAP) increased, and the hepatic portal vein perfusion (HPP) decreased [8–10, 39]. Taken together, these results provided possible directions for future research.

Among numerous liver perfusion parameters, SRRmax, TTP, and MTT are relatively common, sensitive, and accurate indicators of liver microcirculation perfusion resistance; MTT is one of the most sensitive and specific perfusion indicators for the diagnosis of advanced liver fibrosis [36, 37]. TTP reflects the ability of low-molecular weight compounds in plasma to freely enter the Disse cavity, while some investigators demonstrated that TTP in liver fibrosis patients is extended than that in a normal person, which is attributed to sinusoid capillarization and collagen deposition in Disse cavity; also, the extravascular space limits the movement of low-molecular weight compounds [34].

In the current study, PWI perfusion parameters were statistically significant in the following groups (*P* < 0.05): SRRmax values, control group and F3 and F4; TTP values, control group and F2–F4, F1 and F2, F1 and F3, F1 and F4, and F2 and F4; MTT values, control group and F2–F4, F1 and F3, and F1 and F4. This phenomenon indicates that PWI perfusion parameters have a sensitive detection ability for F2–F4 liver fibrosis, especially TTP and MTT values, but the F1 detection ability needs further improvement. The altered trend of the single parameter has a suggestive effect on the detection of F1, while the detection efficiency is significantly improved when the three parameters are combined. The findings of this study also suggested that as the degree of liver fibrosis increased, the SRRmax value decreased, but the TTP and MTT values increased gradually. Intriguingly, with the development of liver fibrosis stage, this change was apparent. The pathological examination showed that different degrees of intrahepatic fiber formation, lobular collapse, distorted and reduced intrahepatic vessels, and increased blood flow resistance decreased the blood flow to the liver parenchyma and slowed the rate of liver perfusion. After injecting the contrast agent, the perfusion curve could visually reflect the trend of slow-in and slow-out of the hepatic fibrosis group, which was related to the sinusoidal capillary vascularization affecting the material exchange between blood and liver cells, thereby resulting in a decrease in blood volume in the liver parenchyma.

Although SRRmax, TTP, and MTT exert a satisfactory predictive effect on early liver fibrosis, sensitivity in the detection of F1 needs further research and improvement. Nevertheless, the present study has some limitations: the sample size is relatively small, the number of rats in F0–F4 is uneven, and the failure rate is slightly high. In future research, we can try to improve the credibility of the experimental results by expanding the sample size. Besides, precise control of drug dosage and proficiency in operational techniques may help to improve the success rate of the experiment. PWI is still in the developmental stage, the technology and model need to be refined further to shorten the imaging time and improve the spatial and temporal resolution, image quality, sensitivity, and accuracy of perfusion parameter analysis [1].

## 5. Conclusions

In conclusion, the current study showed that PWI perfusion parameters (TTP, MTT, and SRRmax) were closely related to liver fibrosis stage (TTP and MTT, positive correlation; SRRmax, negative correlation), especially TTP and MTT values, and the combination of these three parameters significantly improved the detection efficiency of F1. Supposedly, PWI perfusion parameters are predictive indicators of early liver fibrosis, providing an early basis for the clinical intervention and treatment of the condition. These parameters have advantages over conventional imaging techniques and broad application prospects. Hepatic fibrosis can also be studied based on the correlation between the reduction in the number of fenestrations and liver fibrosis stage, which would provide an insight into the pathogenesis of liver fibrosis.

## Figures and Tables

**Table 1:** Liver perfusion parameters between the control group and different degrees of liver fibrosis.

Liver fibrosis stage (*n*)	SRRmax	TTP	MTT
Control (*n* = 8) a	0.754 ± 0.073	14.175 ± 4.845	24.620 ± 5.577
F1 (*n* = 7) b	0.674 ± 0.137	18.433 ± 7.293	28.945 ± 2.758
F2 (*n* = 9) c	0.632 ± 0.154	26.789 ± 3.621 ac, bc	32.502 ± 4.268 ac
F3 (*n* = 11) d	0.603 ± 0.201 ad	31.755 ± 7.308 ad, bd	35.861 ± 4.651 ad, bd
F4 (*n* = 8) e	0.535 ± 0.135 ae	35.213 ± 6.322 ae, be, ce	35.203 ± 5.674 ae, be

Note: SRRmax: ($\overset{—}{x}\pm$ *S*) × 100%; TTP: ($\overset{—}{x}\pm$ *S*)*s*; MTT: ($\overset{—}{x}\pm$ *S*)*s*; lowercase letters a–e: corresponding to the control group, F1–F4 group, meaningful groups are marked in the figure.

**Table 2:** Pearson's correlation analysis between liver perfusion parameters and liver fibrosis stage.

	SRRmax		TTP		MTT	
	*r* value	*P* value	*r* value	*P* value	*r* value	*P* value
Liver fibrosis stage	−0.439	0.003	0.798	*P* < 0.001	0.647	*P* < 0.001

TTP and MTT were correlated with liver fibrosis stage, while SRRmax was low, all *P* < 0.05.

## Data Availability

The data used to support the findings of this study are available from the corresponding author upon request.
